# Association of Suicide Risk With Transition to Civilian Life Among US Military Service Members

**DOI:** 10.1001/jamanetworkopen.2020.16261

**Published:** 2020-09-11

**Authors:** Chandru Ravindran, Sybil W. Morley, Brady M. Stephens, Ian H. Stanley, Mark A. Reger

**Affiliations:** 1 Veterans Integrated Service Network (VISN) 2, Center of Excellence for Suicide Prevention, Canandaigua, New York; 2 Veterans Affairs Puget Sound Health Care System, Seattle, Washington; 3 Department of Psychiatry and Behavioral Sciences, University of Washington, Seattle

## Abstract

**Importance:**

Although interest is high in addressing suicide mortality after the transition from military to civilian life, little is known about the risk factors associated with this transition. To support the ongoing suicide surveillance work of the US Department of Veterans Affairs (VA) Office of Mental Health and Suicide Prevention, examining these factors is important for targeting suicide prevention efforts.

**Objective:**

To examine the prevalence, patterns, and associated characteristics of suicide mortality among US service members after separation from military active status.

**Design, Setting, and Participants:**

This retrospective population-based cohort study obtained demographic and military service data from the VA/Department of Defense Identity Repository. Individuals who served on active duty in the US Army, Navy, Air Force, Marine Corps, or Coast Guard after September 11, 2001, and who separated from active status between January 1, 2010, and December 31, 2017, were included in the cohort. Data analyses were conducted from September 9, 2019, to April 1, 2020.

**Main Outcomes and Measures:**

Suicide mortality within 6 years after separation from military service.

**Results:**

A total of 1 868 970 service members (1 572 523 men [84.1%]; mean [SD] age at separation, 30.9 [9.9] years) separated from the military during the study period. Through the end of the study period (December 31, 2017), 3030 suicides (2860 men and 170 women) were identified as having occurred within 6 years of separation from the military. Statistically significant differences in suicide risk were found by demographic and military service characteristics. Suicide rates after separation were time dependent, generally peaking 6 to 12 months after separation and declining only modestly over the study period. Male service members had a statistically significantly higher hazard of suicide than their female counterparts (hazard ratio [HR], 3.13; 95% CI, 2.68-3.69). Younger individuals (aged 17-19 years; HR, 4.46 [95% CI, 3.71-5.36]) had suicide hazard rates that were approximately 4.5 times higher than those who transitioned at an older age (≥40 years). Service branch remained a risk factor for suicide even 6 years after separation; those who separated from the Marine Corps (HR, 1.55; 95% CI, 1.36-1.78) and the Army (HR, 1.48; 95% CI, 1.31-1.67) had a higher hazard than those who transitioned from the Air Force. The hazard for those who separated from the active component was higher than for those who separated from the reserve component (HR, 1.29; 95% CI, 1.18-1.42). Service members with a shorter length of service had a higher hazard (HR, 1.26; 95% CI, 1.11-1.42) than those with a longer service history.

**Conclusions and Relevance:**

Results of this study show that not all service members who recently transitioned from military life had the same risk of suicide. The data suggest that awareness of military service and demographic characteristics can help identify those most at risk for suicide to target prevention efforts.

## Key Points

**Question:**

What demographic and military service characteristics are associated with suicide risk among service members who recently transitioned from military service?

**Findings:**

In this population-based cohort study of 1 868 970 service members who separated from the military, those who were male, were younger, had shorter length of service, or were separated from the Marine Corps or Army had a higher risk of suicide after separation.

**Meaning:**

Findings of this study suggest that suicide rates increase after transition to civilian life and that awareness of demographic and military service characteristics may help prevent suicide among veterans who are most at risk.

## Teaser

This cohort study investigates the risk factors in suicides that occur after separation from active military service among US veterans.

## Introduction

Rates of suicide in the US military have increased substantially since the early 2000s.^1^ In response, national leaders have dedicated considerable resources to improve the identification and prevention of the factors associated with suicide risk among service members and veterans.^2,3,4,5^ One specific, relevant factor is the transition from military service to civilian life.^5^ Separation from military service is a milestone characterized by a variety of psychosocial stressors and adjustment challenges (eg, disruptions in social support, financial strains, and changes in access to health care and mental health care)^6,7^ that might be associated with increased risk of suicide.

National interest is high in understanding the association of military separation with suicide among service members.^5,8^ The US Department of Veterans Affairs (VA) has issued the 2018 to 2028 National Strategy for Preventing Veteran Suicide, which highlights this critical need among those who have recently transitioned from military service.^5^ Moreover, a 2018 Presidential Executive Order specifically sought to improve suicide prevention services for veterans going back to civilian life.^9^ Despite these national programs, almost no data are available to guide suicide prevention efforts targeted at these particular service members.

Increased risk of suicide has generally been associated with military separation. In a retrospective cohort study of 3.9 million US military personnel who served during Operation Enduring Freedom or Operation Iraqi Freedom, separation from military service was associated with a substantially higher risk of suicide mortality.^10^ These findings converged with the results of a separate analysis of service members in the United States^11^ and a cohort study of Armed Forces personnel in the United Kingdom.^12^ The hazard rate of suicide was about 2.5 times higher for veterans within the first year of separation than for the active duty population.^11,12^ Together, early findings suggest that this transition and the immediate postdischarge period may be a vulnerable time for suicide risk. However, more data are needed to determine which individuals in the transitioning cohort are at high risk and when that risk is highest. To our knowledge, despite the national interest in this problem,^9^ no study has comprehensively examined time-varying associations of suicide with transition to civilian life.

With the goal of guiding the VA’s public health and policy efforts to prevent suicide among US veterans, we conducted a retrospective, population-based cohort study of the prevalence, patterns, and associated characteristics of suicide mortality in service members after separation from active status. In this study, we characterized the factors associated with increased suicide risk among these individuals, building on previous research that demonstrated this group to be at risk. These factors included sociodemographic (eg, sex, race, and ethnicity) and military service (eg, component, branch, and length of service) characteristics.

## Methods

### Study Population

This cohort study was conducted as part of the ongoing quality improvement and operations work of the VA Office of Mental Health and Suicide Prevention and obtained administrative data from the VA/Department of Defense Identity Repository (VADIR).^13^ Analyses were conducted as part of ongoing Veterans Health Affairs operations and program evaluation conducted by the VA Office of Mental Health and Suicide Prevention and therefore did not require IRB review per VA policy.^14^ As such, the study did not require approval from an institutional review board or waiver of informed consent. We followed the Strengthening the Reporting of Observational Studies in Epidemiology (STROBE) reporting guideline.

Administrative records from VADIR included demographic and military service information and were used in this study to identify service members in the active or reserve components of the military who served on active duty. Individuals who served in the US Army, Navy, Air Force, Marine Corps, or Coast Guard after September 11, 2001, and who separated from active status between January 1, 2010, and December 31, 2017, were included in the cohort. Separation referred to either complete separation from military status (discharge) or transition from active status to a reserve component category other than the Selected Reserves (eg, Inactive Ready Reserve).

Demographic characteristics we assessed included age at separation, sex, race, ethnicity, marital status at separation, and educational level at separation. These characteristics were classified using preexisting administrative data. Military service characteristics included the branch, component, and separation date. For service members with multiple transitions, we used the characteristics associated with their latest separation. For analyses, we considered service members as having shorter service experience if they had fewer than 2 years of active duty service in the active component (the minimum active duty contract service requirement) with no other military service or deployment history.

For the core models that included age, sex, race, and ethnicity, the missing data were minimal (<5% of records had missing values) and addressed through listwise deletion. No individuals were lost to follow-up.

### Exposure Variables and Primary Outcome

Separation from active status was the primary exposure variable. Service members entered the cohort on their separation date and exited after 6 years (2190 days), on their date of death, or on December 31, 2017, whichever came first. The time at risk after separation was calculated as the number of days from separation to exiting the cohort. The primary outcome was death from suicide through December 31, 2017. Mortality data were obtained from the VA/Department of Defense Mortality Data Repository, which compiles results from comprehensive searches of the National Death Index. We used *International Statistical Classification of Diseases and Related Health Problems, Tenth Revision* codes X60 to X84, Y87.0, and U03 to identify suicide deaths.

### Statistical Analysis

Crude suicide rates within the 6 years after separation were calculated as the number of suicides that occurred divided by the total person-years at risk across the cohort in the period, and 95% CIs for rates were calculated on the basis of the γ distribution.^15^ Conditional rates were calculated as the number of suicides in the specified time interval divided by the total person-years within the interval for uncensored service members at the start of the interval.

Cox proportional hazards regression models were used to estimate hazard ratios (HRs) and associated 95% profile likelihood CIs for suicides within 6 years of separation for demographic and military service covariates. Bivariate Cox proportional hazards regression models were used to generate unadjusted HRs for each covariate. Adjusted HRs were calculated with multivariable Cox proportional hazards regression models that were adjusted for sex, age group, race, and ethnicity. Statistical significance was identified using 2-tailed Wald χ^2^ tests at a significance level of *P* = .05. All models were checked for Cox proportional hazards regression model assumptions using graphical and analytical methods. Residual analyses were performed to identify the outliers and covariates that introduced instability into the models, and goodness-of-fit tests were performed.

All pair-wise interactions involving age and sex were assessed; these interactions were excluded from primary models because of their negligible impact on HRs and increased instability in the models. The interaction between age and component was statistically significant and was further analyzed using a supplementary model. Data analyses were conducted from September 9, 2019, to April 1, 2020, with SAS Enterprise Guide, version 7.1 (SAS Institute Inc).

## Results

### Primary Analyses

The study cohort comprised 1 868 970 veterans who met the inclusion criteria and contributed 7 047 300 person-years of follow-up time. Among these service members, 1 572 523 were male (84.1%) and 296 447 were female (15.9%), with a mean (SD) age at separation of 30.9 (9.9) years. Most individuals in this cohort were White (1 352 598 [72.4%]) and last served in the Army (889 688 [47.6%]).

Person-years, suicide counts, crude suicide rates, and HRs by demographic and military service characteristics are presented in the Table. Statistically significant differences in suicide risk were found by demographic and military service characteristics. Through the end of the study period (December 31, 2017), 3030 suicides (2860 men and 170 women) were identified. Suicide rates were time dependent, generally peaking 6 to 12 months after separation and declining only modestly over the study period.

**Table.** Association Between Separation From Military Service and Suicide by Demographic Characteristics

Variable	Person-years	No. of suicides within 6 y of separation	Suicide rate per 100 000 (95% CI), %	Unadjusted		Adjusted^a^	
				HR (95% CI)	*P* value	HR (95% CI)	*P* value
Overall	7 047 300	3030	43.0 (41.5-44.6)	NA	NA	NA	NA
Sex							
Male	5 933 608	2860	48.2 (46.5-50.0)	3.15 (2.71-3.70)	<.001	3.13 (2.68-3.69)	<.001
Female	1 113 663	170	15.3 (13.1-17.7)	1 [Reference]	1 [Reference]	1 [Reference]	1 [Reference]
Age at separation, y							
17-19	310 857	211	67.9 (59.0-77.7)	3.83 (3.20-4.58)	<.001	4.46 (3.71-5.36)	<.001
20-24	2 018 071	1246	61.7 (58.4-65.3)	3.49 (3.07-3.98)	<.001	3.62 (3.17-4.15)	<.001
25-29	1 829 395	792	43.3 (40.3-46.4)	2.44 (2.14-2.81)	<.001	2.57 (2.24-2.97)	<.001
30-34	793 464	325	41.0 (36.6-45.7)	2.31 (1.97-2.71)	<.001	2.42 (2.06-2.86)	<.001
35-39	526 081	178	33.8 (29.1-39.2)	1.91 (1.58-2.30)	<.001	2.02 (1.67-2.45)	<.001
≥40	1 569 329	278	17.7 (15.7-19.9)	1 [Reference]	1 [Reference]	1 [Reference]	1 [Reference]
Race							
White	5 069 443	2347	46.3 (44.4-48.2)	1 [Reference]	1 [Reference]	1 [Reference]	1 [Reference]
Black or African American	1 087 787	307	28.2 (25.2-31.6)	0.61 (0.54-0.69)	<.001	0.69 (0.61-0.78)	<.001
Asian, Native Hawaiian, or Pacific Islander	444 267	196	44.1 (38.2-50.8)	0.96 (0.82-1.10)	.55	0.95 (0.82-1.10)	.49
American Indian or Alaskan Native	120 999	68	56.2 (43.6-71.3)	1.22 (0.95-1.53)	.11	1.18 (0.92-1.49)	.18
Ethnicity							
Not Hispanic	6 263 772	2771	44.2 (42.6-45.9)	1 [Reference]	1 [Reference]	1 [Reference]	1 [Reference]
Hispanic	716 788	241	33.6 (29.5-38.2)	0.76 (0.66-0.86)	<.001	0.70 (0.60-0.80)	<.001
Educational level at separation							
Non-high school graduate	971 557	537	55.3 (50.7-60.2)	2.59 (2.14-3.15)	<.001	1.71 (1.41-2.09)	<.001
Standard high school graduate	5 052 402	2301	45.5 (43.7-47.4)	2.13 (1.79-2.56)	<.001	1.32 (1.10-1.59)	.004
Bachelor's degree	607 904	130	21.4 (17.9-25.4)	1 [Reference]	1 [Reference]	1 [Reference]	1 [Reference]
Higher degree	354 986	55	15.5 (11.7-20.2)	0.73 (0.53-0.99)	.046	0.94 (0.67-1.30)	.73
Marital status at separation							
Never married or single	4 984 735	2521	50.6 (48.6-52.6)	2.16 (1.95-2.40)	<.001	1.21 (1.06-1.39)	.006
Married	1 746 505	409	23.4 (21.2-25.8)	1 [Reference]	1 [Reference]	1 [Reference]	1 [Reference]
Divorced, separated, or widowed	177 552	50	28.2 (20.9-37.1)	1.20 (0.89-1.60)	.22	1.43 (1.05-1.92)	.02
Branch							
Marine Corps	1 074 878	679	63.2 (58.5-68.1)	2.35 (2.06-2.68)	<.001	1.55 (1.36-1.78)	<.001
Army	3 370 832	1568	46.5 (44.2-48.9)	1.73 (1.54-1.95)	<.001	1.48 (1.31-1.67)	<.001
Navy	1 239 801	411	33.2 (30.0-36.5)	1.23 (1.07-1.43)	.004	1.05 (0.90-1.22)	.52
Air Force	1 256 880	338	26.9 (24.1-29.9)	1 [Reference]	1 [Reference]	1 [Reference]	1 [Reference]
Coast Guard	104 908	34	32.4 (22.4-45.3)	1.21 (0.83-1.69)	.30	1.09 (0.73-1.56)	.67
Component							
Active	4 920 365	2382	48.4 (46.5-50.4)	1.59 (1.46-1.73)	<.001	1.29 (1.18-1.42)	<.001
Reserve	2 126 935	648	30.5 (28.2-32.9)	1 [Reference]	1 [Reference]	1 [Reference]	1 [Reference]
Length of service							
Shorter service time	788 668	525	66.6 (61.0-72.5)	1.66 (1.51-1.83)	<.001	1.26 (1.11-1.42)	<.001
Standard service time	6 258 632	2505	40.0 (38.5-41.6)	1 [Reference]	1 [Reference]	1 [Reference]	1 [Reference]

Abbreviations: HR, hazard ratio; NA, not applicable.

^a^ Variables were adjusted for sex, age, race, and ethnicity.

Male veterans had a statistically higher hazard of suicide within 6 years of separation (HR, 3.13; 95% CI, 2.68-3.69) compared with female veterans. Hazard generally decreased with increasing age, with younger individuals having hazard rates that were approximately 4.5 times higher than those who transitioned at an older age (17-19 years: HR, 4.46 [95% CI, 3.71-5.36]; 35-39 years: HR, 2.02 [95% CI, 1.67-2.45]), as well as with a higher educational level, with non–high school graduates having the highest hazard (HR, 1.71; 95% CI, 1.41-2.09). Those who were never married (HR, 1.21; 95% CI, 1.06-1.39) or who were divorced, separated, or widowed (HR, 1.43; 95% CI, 1.05-1.92) at the time of transition had a significantly higher hazard than those who were married. Black service members had lower hazard than White service members (HR, 0.69 [95% CI, 0.61-0.78]). Hispanic individuals had a lower hazard than non-Hispanic veterans (HR, 0.70; 95% CI, 0.60-0.80).

**Figure 1.**
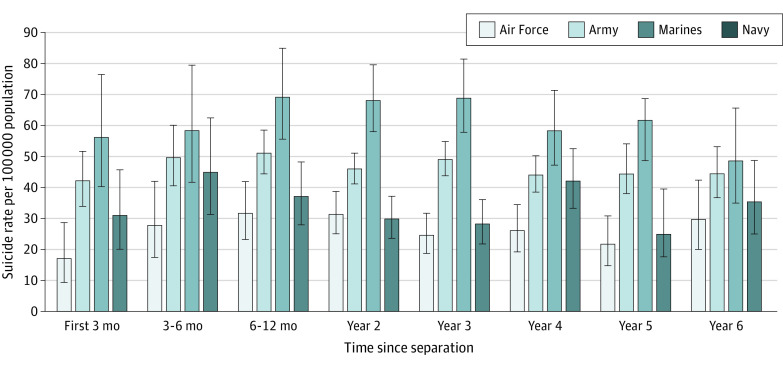
Suicide Rates Conditioned on Time Since Separation by Service Branch With 95% CIs

Figure 1 shows the conditional suicide rates by service branch over the first 6 years after separation. Rates increased during year 1 but peaked at slightly different time points for each branch over the study period. Rates remained stable over the remainder of the 6-year period, with no branch displaying substantial change from year to year. Service members separating from the Marine Corps (HR, 1.55; 95% CI, 1.36-1.78) and Army (HR, 1.48; 95% CI, 1.31-1.67) had a significantly higher hazard (approximately 1.5 times higher) than those separating from the Navy or Air Force. The hazard for former Navy service members (HR, 1.05; 95% CI, 0.90-1.22) was not statistically different from that for Air Force veterans.

**Figure 2.**
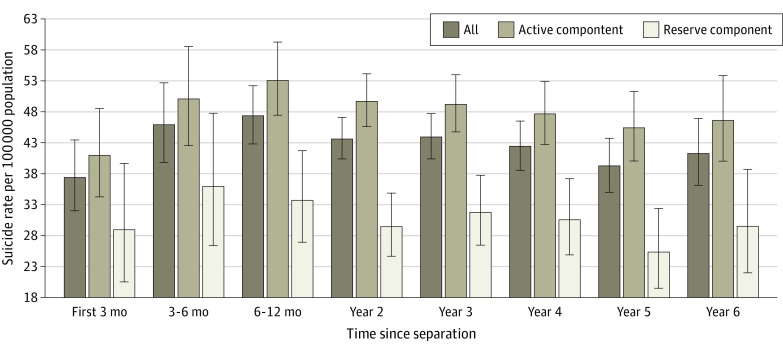
Suicide Rates Conditioned on Time Since Separation by Component With 95% CIs

Conditional suicide rates by component (Figure 2) show similar patterns. The hazard for those who separated from the active component was statistically higher than for those who separated from the reserve component (HR, 1.29; 95% CI, 1.18-1.42). When component interactions with age (dichotomized into younger [<40 years] or older [≥40 years]) were assessed, younger veterans who left the active component had a higher hazard than their younger counterparts who left the reserve component (HR, 1.54; 95% CI, 1.40-1.70). Older service members showed no statistically significant difference in hazard (HR, 0.89; 95% CI, 0.70-1.13). Younger veterans had a significantly higher hazard than older veterans in both components, and this finding was more pronounced for those who had separated from the active component (HR, 3.48; 95% CI, 2.92-4.14) than from the reserve component (HR, 2.00; 95% CI, 1.65-2.43).

**Figure 3.**
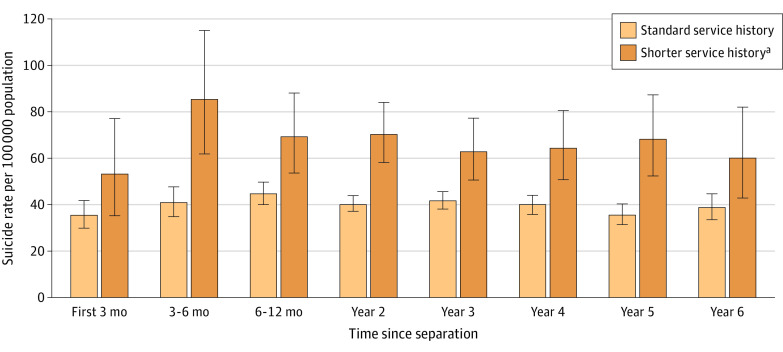
Suicide Rates Conditioned on Time Since Separation by Length of Service With 95% CIs ^a^Shorter service history defined as less than 2 years of service in the active component with no other military service or deployment history.

Service members with a shorter length of service had a statistically higher hazard over the duration of the study period (HR, 1.26; 95% CI, 1.11-1.42) than service members with a more standard service history. Figure 3 shows similar conditional rate patterns over time as those patterns observed when rates were stratified by branch or component. The suicide rate among those whose length of service was fewer than 2 years in the active component increased from the first 3 months of separation, when it was already high (53.2 suicides per 100 000), to 3 to 6 months after transition (85.2 suicides per 100 000) when it peaked.

### Contextualizing Cohort Risk

Separation from active status is a unique characteristic in military populations, and no analog of this characteristic exists with which to compare hazard in nonmilitary populations. To contextualize the general risk in the study cohort, we assessed the standardized mortality ratio, adjusting for sex and age at death compared with other adults in the US during the study period (2010-2017) as identified from the Centers for Disease Control and Prevention WONDER (Wide-ranging Online Data for Epidemiologic Research) databases (standardized mortality ratio, 1.93; 95% CI, 1.87-2.00).

## Discussion

Several investigations have found that veterans have higher suicide rates after transitioning out of the military than service members with active status.^10,11^ To our knowledge, this population-based cohort study was the first to examine specific suicide risk factors by time since transition from active status. We examined demographic and military service characteristics to inform the veteran suicide prevention efforts of the VA.

This study showed that suicide rates followed a nuanced pattern after individuals left the military. The highest suicide rates were observed in year 1, but they increased over the first 6 months and generally peaked in the 6 to 12 months after separation. This pattern was true for service members who left the Army, Marine Corps, or Air Force; the rates for those who last served in the Navy peaked 3 to 6 months after their transition. Previous research has suggested that, after transition, veterans can face challenges in a variety of areas, including employment, finances, mental health, access to health care, and social support.^6,7^ Therefore, we speculate that psychosocial stressors would increase in year 1 after separation, contributing to the patterns observed. In support of this conjecture, we found that, compared with recently transitioned service members with a bachelor’s degree, those with fewer years of education (ie, high school or non–high school graduate) had a higher hazard of suicide. These individuals may have had a more difficult time finding postmilitary employment, thereby exacerbating their financial concerns, difficulties establishing health care, and attendant psychosocial stressors (and, therefore, suicide risk).

Alternatively, preenlistment characteristics, experiences during military service, or experiences after separation may contribute to these findings. The present study was not designed to examine whether separation from the military was associated with suicide; previous population-based research has already shown that the hazard rate of suicide was approximately 2.5 times higher for veterans within the first year of separation compared with service members on active duty.^11^ Study findings suggested that, within the high-risk cohort of transitioning service members, suicide prevention resources are especially important in the first year and remain important for at least 6 years given that the rates did not decline substantially within the study period. This finding was consistent with the finding from another study^11^ that examined suicide rates in the years after transition.

Suicide rates after transition differed substantially by service branch. Many studies have reported the differences in suicide rates by branch during active duty service; typically, the Army and Marine Corps had higher rates compared with the rates of the Navy and Air Force.^16^ To our knowledge, this study was the first to examine rates by service branch in the first 6 years after separation from military life. The data suggested that service branch continues to represent a substantial risk factor for suicide. Suicide hazard rates for veterans of the Army and Marines Corps were approximately 1.5 times higher than the rates for Air Force (or Navy) veterans. Compared with the other branches, the Marines Corps had the highest crude suicide rate every year after transition. It is unclear which factors were associated with these rate differences. Each branch has a unique culture, training experiences, deployment experiences, and demographic compositions. The branches may also recruit service members who are unique in some ways. Many veterans continue to strongly identify with their branch even after leaving the service. Suicide research and suicide prevention efforts for veterans almost never consider the branch, and once a service member is separated from service, that individual is typically considered a veteran.^5,17^ These results suggest that service branch should be considered a military risk factor for suicide, even after the transition to civilian life.

Military component was another risk factor during the transition period. Many reservists spend only 1 weekend a month and 2 weeks a year in training. Concerns have been raised about the preparation and resources provided to reservists, especially for deployment.^18^ Previous research has highlighted some of the unique mental health challenges among reservists vs those serving in the active component,^19^ and concerns have been raised periodically about suicide rates among reservists.^20,21^ However, results from this study showed that reservists had a lower risk of suicide after separating from the military than those who left the active component. We speculated that citizen soldiers in the reserve component, who often maintain civilian employment and considerable ties to their civilian life, would experience fewer transition adjustments, but this possibility requires additional study.

The high suicide rate seen in year 1 after separation was especially prominent among those whose length of service was fewer than 2 years in the active component. This group’s suicide rates increased from the first 3 months when it was already high (53.2 suicides per 100 000) to 3 to 6 months after transition (85.2 suicides per 100 000) when it peaked. Service members who served fewer than 2 years likely had an atypical end to their military service (eg, injury, medical condition, mental health problem, discipline issue, or legal issue); however, we did not have access to data that characterized the nature of this group’s service. Service members with an atypical service history likely encounter unique challenges after the transition to civilian life. For example, mental health problems that led to a military discharge may be associated with challenges in social functioning, social support, occupational functioning, and personal finances.^6,7^ These challenges may increase the risk of suicide, but these associations need further examination.

Results of this study also suggest that not all demographic groups who separated from military service were at equal risk. Young individuals (aged 17-19 years) had suicide hazard rates that were almost 4.5 times higher than those who transitioned at an older age (≥40 years). We were unable to determine whether the high suicide rates among the younger veterans were directly associated with the return to civilian life or with the demographic characteristics associated with suicide risk. However, among the cohort of transitioning service members, younger individuals were at substantially elevated risk compared with older veterans. The cohort with the shorter length of service had suicide rates similar to the rates of the youngest age group of service members and overlapped substantially with this group. However, even among veterans in the 25-to-29-year age group (ie, less likely to have an atypical end to their military career), the suicide hazard rates were more than 2.5 times higher than in older veterans after adjusting for other demographic characteristics. Although it is intuitive to hypothesize that younger service members would experience more transition-adjustment challenges (eg, difficulty finding a job; less social or family support), more research is needed to determine why younger veterans have an elevated risk of suicide.

The other demographic risk factors we found were similar to risk factors commonly observed in epidemiological studies of suicide.^22^ Non-Hispanic White male veterans with less education and divorced individuals had higher suicide rates than their respective demographic comparison groups. Therefore, these traditional suicide risk factors are relevant to prevention efforts targeted at service members who have left the military and returned to civilian life.

### Strengths and Limitations

This study has some strengths. Among these strengths were the analysis of suicide by time since transition and the use of a large cohort of US veterans.

This study also has some limitations. First, we lacked data to further characterize risk factors for the transitioning service members that we analyzed; these data included medical and mental health data and demographic changes after the separation. Second, the potential for variability in the manner in which coroners and medical examiners ascertained death is a recognized limitation of death certificate records in general.^23^ However, based on additional analyses we conducted, including deaths of undetermined intent and suicides combined as the outcome, we found negligible changes to HRs and statistical significance.

## Conclusions

National leaders at the highest levels of the US government have been concerned about suicide rates among service members transitioning to civilian life. We believe this cohort study provides much-needed data to help inform prevention efforts among this veteran cohort. Suicide rates increased in the early months after transition, peaked by the end of the first year, and remained high for years to come. Prevention efforts may be helpful for younger service members with fewer than 2 years of military service. Furthermore, service branch remains a risk factor for many years after transition and could be examined for more focused suicide prevention efforts.
